# Prognostic model for the exemption of adjuvant chemotherapy in stage IIIC endometrial cancer patients

**DOI:** 10.3389/fendo.2022.989063

**Published:** 2022-10-26

**Authors:** Xi-Lin Yang, Feng-Leng Yang, Ling-Na Kou, Da-Jun Wu, Cong Xie

**Affiliations:** ^1^ Department of Radiation Oncology, Chengdu Women’s and Children’s Central Hospital, School of Medicine, University of Electronic Science and Technology of China, Chengdu, China; ^2^ Department of Radiology, Chengdu Women’s and Children’s Central Hospital, School of Medicine, University of Electronic Science and Technology of China, Chengdu, China; ^3^ Department of Medical Oncology, Sichuan Cancer Hospital and Institute, Chengdu, China; ^4^ Department of Gynecology, Chengdu Women’s and Children’s Central Hospital, School of Medicine, University of Electronic Science and Technology of China, Chengdu, China

**Keywords:** endometrial cancer, stage IIIC, nomogram, adjuvant radiotherapy, adjuvant chemotherapy

## Abstract

**Background:**

This study aimed to develop a nomogram to predict the survival for stage IIIC endometrial cancer (EC) patients with adjuvant radiotherapy (ART) alone and personalize recommendations for the following adjuvant chemotherapy (ACT).

**Methods:**

In total, 746 stage IIIC EC patients with ART alone were selected from the Surveillance, Epidemiology, and End Results (SEER) registry. Cox regression analysis was performed to identify independent risk factors. A nomogram was developed accordingly, and the area under the receiver operating characteristic curve (AUC) and C-index were implemented to assess the predictive power. The patients were divided into different risk strata based on the total points derived from the nomogram, and survival probability was compared between each risk stratus and another SEER-based cohort of stage IIIC EC patients receiving ART+ACT (cohort ART+ACT).

**Results:**

Five independent predictors were included in the model, which had favorable discriminative power both in the training (C-index: 0.732; 95% CI: 0.704–0.760) and validation cohorts (C-index: 0.731; 95% CI: 0.709–0.753). The patients were divided into three risk strata (low risk <135, 135 ≤ middle risk ≤205, and high risk >205), where low-risk patients had survival advantages over patients from cohort ART+ACT (HR: 0.45, 95% CI: 0.33–0.61, *P* < 0.001). However, the middle- and high-risk patients were inferior to patients from cohort ART+ACT in survival (*P* < 0.001).

**Conclusion:**

A nomogram was developed to exclusively predict the survival for stage IIIC EC patients with ART alone, based on which the low-risk patients might be perfect candidates to omit the following ACT. However, the middle- and high-risk patients would benefit from the following ACT.

## Introduction

Endometrial cancer (EC) is the most prevalent gynecological malignancy in developed countries, with 65,950 new cases expected and 12,550 deaths in the United States alone in 2022 (1). EC patients with lymph node (LN) metastases, which have been relocated as stage IIIC in the revised 09 International Federation of Gynecology and Obstetrics (FIGO) staging system, still accounted for approximately 8–10% of all EC cases (2, 3), though most of the EC patients were diagnosed at a relatively early stage. The recommended primary treatment for stage IIIC patients was surgery, consisting of total hysterectomy, bilateral salpingo-oophorectomy, and systematic lymphadenectomy, and adjuvant treatment would be delivered depending on the pathological assessment. However, the optimal adjuvant treatment for these patients is still open to debate given that stage IIIC EC is a significantly heterogenous disease with a 5-year overall survival (OS) that ranges from 40 to 80% (4–6).

As one of the most prominent randomized clinical trials comparing different adjuvant treatment modalities for stage III EC patients, the PORTEC-3 trial failed to specifically address whether combined adjuvant chemotherapy with radiotherapy (ACT+ART) was a better treatment choice for stage IIIC patients over adjuvant radiotherapy (ART) alone (7). Several retrospective studies have established the advantages of ACT+ART over ART alone for post-operative stage IIIC EC patients (8–10). However, the subgroup analysis in Chapman’s research demonstrated the absence of a significant difference between ART+ACT and ART alone in stage IIIC EC patients with a grade 1/2 endometrioid disease (8), which was in tune with the study from Binder et al. showing that low-grade patients would benefit similarly from adjuvant ART+ACT or ART alone (9). Moreover, we have noticed that a study from Taiwan (China) indicated that there was no survival difference between ART+ACT and ACT alone in stage IIIC EC patients when the total positive lymph node number did not exceed 5 (11), which further highlighted the value of the lymph node status in guiding the adjuvant treatment.

The lack of a reliable model incorporating all of these pathological indices to predict which individuals would benefit from ART alone while avoiding overtreatment impelled us to construct this model to better guide the adjuvant treatment for stage IIIC EC patients.

## Methods

### Selection of patients

We retrieved the data of EC patients diagnosed between 2010 and 2017 from the Surveillance, Epidemiology, and End Results (SEER) database which captured approximately 34.6% of the cancer statistics in the United States (12), and a case listing was formed through the SEER*Stat software (version 8.4.0; http://seer.cancer.gov/seerstat/). Exemption for ethic review was obtained due to the de-identified feature of the information collected from this public database.

All pathologically stage IIIC EC patients treated with primary surgery—including total hysterectomy, bilateral salpingo-oophorectomy, systematic lymphadenectomy, and ART alone—were included, and the exclusion criteria were as follows: (I) T4 or M1 stage, (II) patients with neoadjuvant radiotherapy, (III) patients who received ACT alone or ACT+ART, (IV) patients who succumbed to surgery complications (survival time of less than 1 month), and (V) patients with insufficient information. It is worth noting that the detailed selection process is listed in **Supplementary Table S1**.

### Cohort definition and study covariate

Patients’ data regarding the year of diagnosis, age, marital status, race, grade (differentiation), histology type, T stage, FIGO stage, tumor size, number of retrieved LN, number of positive LN, survival time, and survival status were collected. It is noteworthy that rare pathological histology type was not included in this study, and we presented the histology type as type I (endometrioid cancer and adenocarcinoma) and type II consisting of serous cancer, carcinosarcoma, and clear cell cancer. The log odds of positive lymph nodes (LODDS), defined as log_e_[(number of positive LN + 0.5)/(number of negative LN + 0.5)], was calculated to represent the LN status given that our previous studies have demonstrated that LODDS was the optimal LN index to predict the survival probability for EC patients with LN metastases (13, 14). X-tile software (version 3.6.1; Yale University, New Haven, CT, USA) was used to identify the best cutoff values for continuous variables including age, tumor size, and LODDS given the inconsistency of these cutoff values from previous research (8, 15–18). As a result, age was categorized as ≤55, 56–75, and ≥76, and tumor size was labeled as ≤3.5, 3.6–5.0, and ≥5.1 cm. Similarly, the LODDS was divided into three subgroups (LODDS1: ≤–0.96, LODDS2: -0.96 to 0.55, and LODDS3: ≥0.56). The remaining variables were also displayed as the year of diagnosis (2010–2013 and 2014–2017), race (White, Black, and Asian/Alaska Indian), marital status (married, separated, and never married or unmarried), grade/differentiation (I/well, II/moderate, and III+IV/poor+un), T stage (T1a, T1b, T2, T3a, and T3b), and FIGO stage (IIIC1 and IIIC2). Lastly, we randomly split these patients into the training and validation cohorts with a ratio of 7:3 as indicated by the previous studies (19, 20).

### Statistical considerations

The categorical variables in this study were estimated as percentages or frequencies and compared using Pearson *χ* ^2^ test or Fisher’s exact test. Univariate and multivariate Cox regression analyses were applied to identify the independent risk factors for OS in the training cohort, and a predicting nomogram incorporating these risk factors was constructed accordingly. The internal validation of this model consisted of two steps. Firstly, C-index, calculated by bootstrapping, was used to evaluate the discriminative ability of this model, which varied from 0.5 to 1.0, with 0.5 indicating random chance and 1.0 representing perfect fit. Secondly, calibration curves were plotted to visualize the relationship between the predictive and observed outcomes, where the closer the predictive curve to the observed curve, the better predictive accuracy the model had (21). Moreover, the validation cohort was used to externally validate these results. The receiver operating characteristic curve was plotted to further visualize the ability of the model in predicting the 1-, 3-, and 5-year OS in both of the training and validation cohorts.

To further explore the clinical value of this model, we stratified the patients from the training cohort into three risk groups (low risk, middle risk, and high risk) based on the total points derived from the nomogram, and the OS curves in different risk strata were plotted. Subsequently, the survival probability between each risk strata and another SEER-based cohort of stage IIIC EC patients receiving both ART+ACT (cohort ART+ACT) was compared using the Kaplan–Meier method. As a result, the candidates for ART alone would be identified. Given that our prediction model was developed merely based on the patients with ART alone and that the applicability of this model for patients with ART+ACT was uncertain, the patients from cohort ART+ACT were not further stratified.

All analyses were operated *via* R software (version.3.6.1; http://www.r-project.org). A two-tailed *P*-value <0.05 was recognized as statistically significant.

## Results

### Characteristics of patients

After selection, we identified 746 stage IIIC EC patients with ART alone from the database, and the median follow-up time for these patients was 61 months (interquartile range: 29–93 months). The sample size was considerably enough to develop a prediction model considering that at least 10 events were needed for each variable (22). Patients aged between 56 and 75 (69.7%) and with white ethnicity (72.8%), grade III/IV (68.4%), and histological type I (64.6) accounted for a big proportion of the training cohort (*N* = 522), where patients characterized with tumor size ranging from 3.6 to 5.0 cm (52.7%) and patients with LODDS1 (LODDS ≤-0.96) (61.9%) were more than half of this cohort. Patients with stage IIIC1 disease also accounted for a bigger proportion than those with stage IIIC2 disease in the training cohort (57.7 *vs*. 42.3%) (**Table 1**). No significant difference was observed in terms of the baseline characteristics between the training and validation cohorts (all *p >*0.05) (**Table 1**).

**Table 1:** Demographic and clinico-pathological characteristics in the training and the validation cohorts.

Variables	Training cohort (*N* = 522)	Validation cohort (*N* = 224)	*P*-value
Year diagnosed			0.530
2010–2013	230 (44.1%)	105 (46.9%)	
2014–2017	292 (55.9%)	119 (53.1%)	
Age			0.215
≤55	93 (17.8%)	42 (18.8%)	
56–75	364 (69.7%)	144 (64.3%)	
≥76	65 (12.5%)	38 (16.9%)	
Marital status			0.665
Married	242 (46.4%)	99 (44.2%)	
Separated	163 (31.2%)	68 (30.4%)	
Never married	117 (22.4%)	57 (25.4%)	
Race			0.491
White	380 (72.8%)	163 (72.8%)	
Black	85 (16.3%)	31 (13.8%)	
Asian/Alaska Indian	57 (10.9%)	30 (13.4%)	
Grade (differentiation)			0.339
I (well)	55 (10.5%)	29 (12.9%)	
II (moderate)	110 (21.1%)	54 (24.1%)	
III/IV (poor/un)	357 (68.4%)	141 (63.0%)	
Histology			0.190
Type I	336 (64.4%)	156 (69.6%)	
Type II	186 (35.6%)	68 (30.4%)	
T stage			0.700
T1a	87 (16.7%)	44 (19.6%)	
T1b	78 (14.9%)	31 (13.8%)	
T2	170 (32.5%)	63 (28.2%)	
T3a	122 (23.4%)	56 (25.0%)	
T3b	65 (12.5%)	30 (13.4%)	
Tumor size (cm)			0.469
≤3.5	140 (26.8%)	57 (25.4%)	
3.6–5.0	275 (52.7%)	112 (50.0%)	
≥5.1	107 (20.5%)	55 (25.6%)	
09 FIGO stage			0.287
IIIC1	301(57.7%)	119 (53.1%)	
IIIC2	221(42.3%)	105 (46.9%)	
LODDS			0.535
LODDS1	323 (61.9%)	140 (62.5%)	
LODDS2	139 (26.6%)	53 (23.7%)	
LODDS3	60 (11.5%)	31 (13.8%)	

FIGO, Federation International of Gynecology and Obstetrics; LODDS, log odds of positive lymph node.

### Identification of independent risk factors

We chose age, race, grade, histology, T stage, tumor size, and LODDS from the univariate Cox analysis (all *p <*0.05). Surprisingly or not, 09 FIGO stage was not able to discern the patients with optimal overall survival from those who had not (*p =* 0.062), which was in accordance with the previous studies (9, 13, 14, 16, 18). Multivariate analysis was sequentially performed to identify the age, grade, T stage, tumor size, and LODDS as the independent risk factors for the survival outcomes in stage IIIC EC patients with ART alone (**Table 2**).

**Table 2:** Univariate and multivariate Cox regression analysis on variables for the prediction of overall survival in the training cohort (*N* = 522).

	Univariate analysis			Multivariate analysis		
Variable	HR	95% CI	*P*-value	HR	95% CI	*P*-value
Year diagnosed						
2010–2013	Ref	Ref				
2014–2017	0.906	(0.722–1.136)	0.391			
Age						
≤55	Ref	Ref		Ref	Ref	
56–75	2.050	(1.420–2.960)	**<0.001**	2.464	(1.676–3.623)	**<0.001**
≥76	3.047	(1.983–4.682)	**<0.001**	3.333	(2.123–5.233)	**<0.001**
Marital status						
Married	Ref	Ref				
Separated	1.238	(0.964–1.590)	0.094			
Never married	0.735	(0.536–1.009)	0.057			
Race						
White	Ref	Ref				
Black	1.620	(1.221–2.149)	**<0.001**			
Asian/Alaska Indian	1.025	(0.713–1.473)	0.895			
Grade (differentiation)						
I (well)	Ref	Ref		Ref	Ref	
II (moderate)	2.018	(0.934–4.360)	0.074	2.107	(0.970–4.578)	0.059
III/IV (poor/un)	7.065	(3.478–14.350)	**<0.001**	6.061	(2.930–12.536)	**<0.001**
Histology						
Type I	Ref	Ref				
Type II	2.012	(1.603–2.526)	**<0.001**			
T stage						
T1a	Ref	Ref		Ref	Ref	
T1b	0.844	(0.566–1.260)	0.407	1.049	(0.670–1.643)	0.835
T2	1.072	(0.696–1.652)	0.752	1.184	(0.813–1.725)	0.379
T3a	1.746	(1.187–2.568)	**0.005**	1.444	(1.011–2.063)	**0.044**
T3b	2.175	(1.429–3.311)	**<0.001**	1.537	(1.030–2.295)	**0.036**
Tumor size (cm)						
≤3.5	Ref	Ref		Ref	Ref	
3.6–5.0	1.612	(1.195–2.174)	**0.002**	1.490	(1.100–2.026)	**0.011**
≥5.1	1.973	(1.404–2.772)	**<0.001**	1.800	(1.254–2.583)	**0.001**
09 FIGO stage						
IIIC1	Ref	Ref				
IIIC2	1.241	(0.990–1.555)	0.062			
LODDS						
LODDS1	Ref	Ref		Ref	Ref	
LODDS2	1.852	(1.432–2.394)	**<0.001**	1.504	(1.150–1.967)	**0.003**
LODDS3	3.357	(2.465–4.570)	**<0.001**	2.812	(2.007–3.941)	**<0.001**

HR, hazard ratio; CI, confidence interval; LODDS, log odds of positive lymph node.

Bold values denote statistical significance at the P < 0.05 level in the univariate and multivariate analysis.

### Development and validation of the nomogram

**Figure 1:**
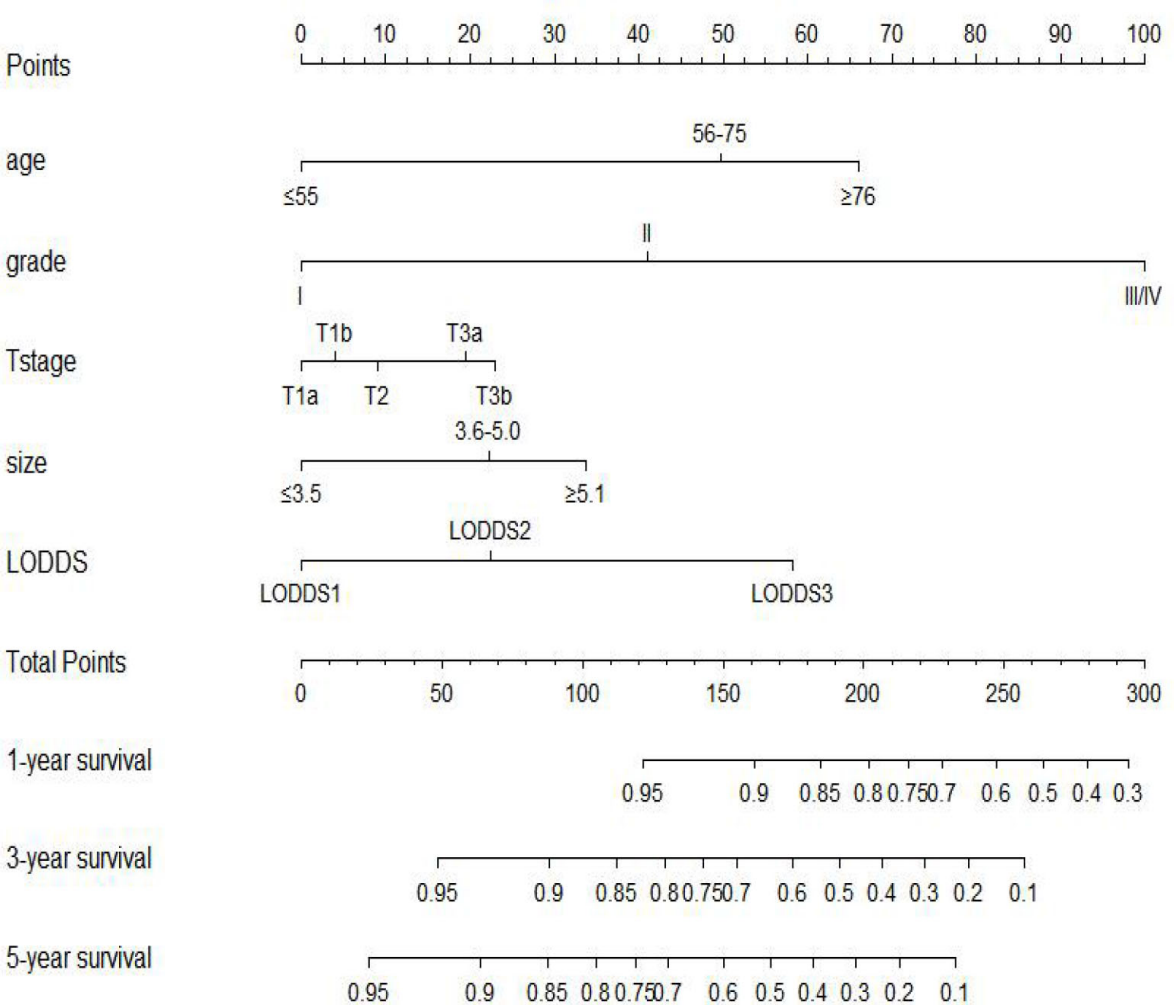
Prognostic nomogram to predict 1-, 3-, and 5-year overall survival in stage IIIC endometrial cancer patients with adjuvant radiotherapy alone.

**Figure 2:**
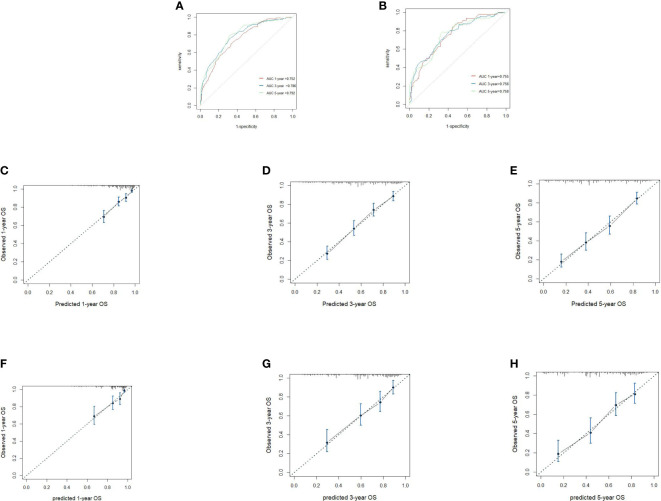
Receiver operating characteristics to describe the predictive power of the model in the training cohort **(A)** and the validation cohort **(B)**. Calibration curves of 1-year **(C)**, 3-year **(D)** and 5-year overall survival (OS) **(E)** for stage IIIC endometrial cancer patients with adjuvant radiotherapy alone in the training cohort. Calibration curves of 1-year **(F)**, 3-year **(G)**, and 5-year OS **(H)** in the validation cohort.

We included all of the significant predictors identified in the multivariate Cox regression analysis to build this original model (**Figure 1**), which yielded a Harrell’s C-index of 0.732 (95% CI: 0.704–0.760) and was able to effectively predict the 1-, 3-, and 5-year OS for stage IIIC EC patients with ART alone (all AUC values >0.700) (**Figures 2A, B** **)**. The calibration curves of the model were then plotted to visualize the optimal consistency between the model-predicted and observed survival probability in the training cohort (**Figures 2C–E**). To validate the predictive power of this model externally, we applied the validation cohort, which turned out to possess a resembling C-index of 0.731 (95% CI: 0.709–0.753). Besides this, decent consistency was observed in the calibration curves of the validation cohort, further underpinning the predictive accuracy of this model (**Figures 2F–H**).

### Clinical utility of the model

**Figure 3:**
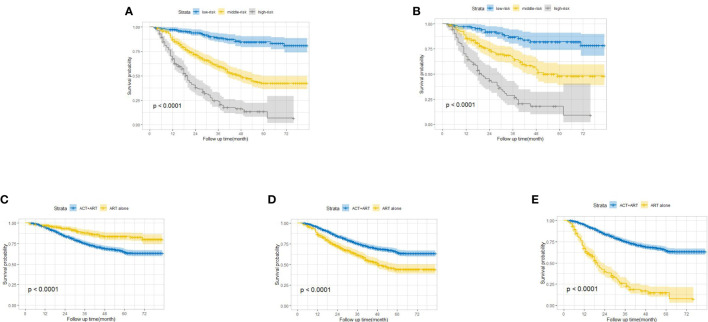
Kaplan–Meier overall survival curves for stage IIIC endometrial cancer patients with adjuvant radiotherapy alone stratified by different risks in the training cohort **(A)** and the validation cohort **(B)**. Comparison of survival probability between low-risk patients and patients from cohort ART+ACT **(C)**, middle-risk patients and patients from cohort ART+ACT **(D)**, and high-risk patients and patients from cohort ART+ACT **(E)**.

Apart from the ability to project the survival outcomes for stage IIIC EC patients with ART alone, we stratified the patients into three distinct risk strata according to the total points derived from the nomogram to further explore the clinical utility of this model. Overt separation among the OS curves belonging to each risk group (low risk <135, 135 ≤ middle risk ≤205, and high risk >205) was presented in both the training and validation cohorts (*p <* 0.0001) (**Figures 3A, B** **)**. Furthermore, we compared the survival probability between the patients from each risk group and the patients from cohort ART+ACT (the baseline characteristics of these patients are documented in **Supplementary Table S2**). Compared with the patients from cohort ART+ACT, patients in the low-risk group were associated with a decreased risk (HR = 0.45, 95% CI: 0.33–0.61, *P* < 0.001) (**Figure 3C**). However, the patients from cohort ART+ACT possessed survival advantages over the patients in the middle-risk group (HR = 1.87, 95% CI: 1.58–2.22, *P* < 0.001) and the high-risk group (HR = 5.40, 95% CI: 4.47–6.53, *P* < 0.001) (**Figures 3D, E** **)**. In summary, the patients benefitting most from ART alone were the nomogram-generated low-risk patients, who might be exempted from ART+ACT without compromising the survival probability. On the contrary, more intensive adjuvant treatment was required for the middle- and high-risk patients.

## Discussion

It has been widely acknowledged that the primary treatment for the EC patients with LN metastases was radical surgery, while the optimal adjuvant treatment modality after operation for these patients remained controversial, which led to the ambiguous recommendation for post-operative stage IIIC EC patients as external beam radiotherapy ± vaginal brachytherapy ± systemic therapy in the National Comprehensive Cancer Network guidelines (23). Two well-known prospective randomized trials have tried to compare the adjuvant treatment efficacy between single modality and combined modality in stage III EC patients (7, 24). However, the PORTEC-3 trial failed to draw an exclusive conclusion on whether combined modality was better than single modality in stage IIIC EC patients, and the GOG-258 trial appeared to conflict with some real-world reports where the single modality seemed to be inferior to the combined modality (8–10). Therefore, a tailored adjuvant treatment on the basis of the characteristics of each individual was required.

In the process of selecting the predictors, the role of histology type drew much of our attention, which showed a significant impact on overall survival in the univariate analysis (*P* < 0.001) but did not hold its position in the multivariate analysis (*P* = 0.287). Despite the consensus that type II EC was a more aggressive disease than type I EC (25), this index indeed loses the ability to differentiate the survival outcomes in stage IIIC EC patients with ART alone. The same phenomenon was also observed by previous investigators who found that stage IIIC EC patients with the same grade would have an equivalent survival probability from different treatment modalities despite the histology type (8, 9), which implied that grade clearly had a much bigger role to play compared with histology type in differentiating the survival outcomes for stage IIIC EC patients. Besides this, grade had the greatest length in our nomogram (**Figure 1**), indicating the strongest prediction ability, which further proved that the predictive power of histology type would be diminished when it was adjusted together with the grade. Other emerging predictive biomarkers including molecular subtypes were not under evaluation in our study given the lack of large-scale randomized clinical trials to establish the role of these subtypes in treatment decision-making (8, 26, 27). The five predictors incorporated in our model did not exceed our initial anticipation, which were consistent with some previous studies (11, 17, 28, 29). However, none of the previous studies had evaluated the predictive power of the combination of these indices in stage IIIC EC patients with ART alone, let alone guide the clinical decision-making using this combination.

As one nascent calculation method integrating pivotal clinical–pathological risk factors to predict oncological outcomes, nomogram has been widely used in EC patients (14, 30–32). however, few of these had focused exclusively on stage IIIC EC patients (14). Actually, we have developed a pretty solid prediction model for post-operative stage IIIC EC patients receiving different kinds of adjuvant treatment in previous research (14), which was able to individually predict the oncological outcomes for stage IIIC EC regardless of the adjuvant treatment modality, but it was not suitable for adjuvant treatment decision-making. Therefore, we further constructed this prediction model where only stage IIIC EC patients with ART alone were included. Beyond prediction for individuals’ outcome, the most novel feature of this model was that it possessed the potential to guide the adjuvant treatment for post-operative stage IIIC EC patients, which further expanded the scope of its clinical utility. Several previous studies have explored the optimal sequence of the ART and ACT. However, a consensus has not been reached (33–35), where concurrent chemoradiation, ART sequenced with ACT, or upfront ACT did not seem to impact the survival outcomes for stage IIIC EC patients. Despite this, ART delivered within 8 weeks after surgery has been strongly recommended (36, 37). Therefore, few of the cancer institutions would prefer upfront ACT before ART, which further reinforced the clinical applicability of our model—that is, all of the stage IIIC EC patients after ART could be assessed by the model to determine whether sequential CT should be delivered or not. There was a beneficial trend for nomogram-generated low-risk patients with ART alone rather than ART+ACT, although we just simply compared the survival difference between the low-risk group and patients receiving ART+ACT.

Some limitations in this study had to be noted. Selection bias in terms of diagnostic methods, censorship, or follow-up arrangement was hard to avoid given the retrospective nature of this study. With the well-known limitation of the SEER database, we were unable to retrieve some important information such as the lympho-vascular space invasion status and the sequence of ACT and ART, which might impair the discriminative ability of our model in some ways. Our prediction model would surely be tested in different datasets across all sorts of institutions in the future, with which some revisions might be imparted to make this model become more accurate. However, our design philosophy for this model might be extrapolated to some other malignancies with uncertain recommendations for the treatment choices.

## Conclusion

A nomogram was constructed and validated to individually predict the survival probability for stage IIIC EC patients with ART alone and potentially tailor the adjuvant treatment for these patients. In this study, nomogram-generated low-risk patients were candidates for omitting of the sequential ACT; however, middle- and high-risk patients might benefit from ACT following the ART.

## Data availability statement

Publicly available datasets were analyzed in this study. This data can be found here: https://seer.cancer.gov/data/.

## Author contributions

Conception/design: X-LY, CX, and D-JW. Provision of study materials or patients: X-LY. Collection and/or assembly of data: X-LY, L-NK, and F-LY. Data analysis and interpretation: X-LY, L-NK, and F-LY. Manuscript writing: X-LY. Manuscript revision: F-LY, CX, and D-JW. Final approval of the manuscript: all authors. All authors contributed to the article and approved the submitted version.

## Acknowledgments

We would like to thank those who have been involved in the establishment of the SEER database and making this database available online.

## Conflict of interest

The authors declare that the research was conducted in the absence of any commercial or financial relationships that could be construed as a potential conflict of interest.

## Publisher’s note

All claims expressed in this article are solely those of the authors and do not necessarily represent those of their affiliated organizations, or those of the publisher, the editors and the reviewers. Any product that may be evaluated in this article, or claim that may be made by its manufacturer, is not guaranteed or endorsed by the publisher.
