# Supplementary material for: Prognostic model for the exemption of adjuvant chemotherapy in stage IIIC endometrial cancer patients
Source: Front Endocrinol (Lausanne). 2022 Oct 26;13:989063. doi: 10.3389/fendo.2022.989063 (PMC9643711; doi:10.3389/fendo.2022.989063)
Supplement: Supplementary file 1 [file Table_1.docx]

| **Table.S1** The selection process for eligible patients. | | | |
| --- | --- | --- | --- |
| Steps | Inclusion Criteria | N.of excluded patients | N.of remaining patients |
| 1 | Patients diagnosed with endometrial cancer between 2010 and 2017 | - | 112868 |
| 2 | Patients with LN metastasis | 87100 | 25768 |
| 3 | Patients with T1-T3 stage | 7596 | 18172 |
| 4 | Patients without distant metastases (M0 stage) | 7395 | 10777 |
| 5 | Patients with at least total hysterectomy | 4395 | 6382 |
| 6 | Patients with lymphadenectomy | 2766 | 3616 |
| 7 | Patients didn’t recieve neoadjuvant radiation therapy | 619 | 2997 |
| 8 | Patients diagnosed with common pathological type* | 541 | 2456 |
| 9 | Patients didn’t die from surgical complications^※^ | 87 | 2369 |
| 10 | Patients recieved adjuvant radiotherapy alone | 1464 | 905 |
| 11 | Patients with sufficient information | 159 | 746 |

LN: lymph node.

*Type Ⅰ including endometrioid and adenocarcinoma and type Ⅱ including serous, carcinosarcoma and clear cell carcinoma.

^※^Patients’ survival time less than 1 month was difined as succumbing to surgical complications.
